# A Critical Appraisal of Solubility Enhancement Techniques of Polyphenols

**DOI:** 10.1155/2014/180845

**Published:** 2014-03-03

**Authors:** Harkiran Kaur, Gurpreet Kaur

**Affiliations:** Department of Pharmaceutical Sciences and Drug Research, Punjabi University, Patiala, Punjab 147002, India

## Abstract

Polyphenols constitute a family of natural substances distributed widely in plant kingdom. These are produced as secondary metabolites by plants and so far 8000 representatives of this family have been identified. Recently, there is an increased interest in the polyphenols because of the evidence of their role in prevention of degenerative diseases such as neurodegenerative diseases, cancer, and cardiovascular diseases. Although a large number of drugs are available in the market for treatment of these diseases, however, the emphasis these days is on the exploitation of natural principles derived from plants. Most polyphenols show low *in vivo* bioavailability thus limiting their application for oral drug delivery. This low bioavailability could be associated with low aqueous solubility, first pass effect, metabolism in GIT, or irreversible binding to cellular DNA and proteins. Therefore, there is a need to devise strategies to improve oral bioavailability of polyphenols. Various approaches like nanosizing, self-microemulsifying drug delivery systems (SMEDDS), microencapsulation, complexation, and solid dispersion can be used to increase the bioavailability. This paper will highlight the various methods that have been employed till date for the solubility enhancement of various polyphenols so that a suitable drug delivery system can be formulated.

## 1. Introduction

Naturally occurring active moieties have been used in therapy since ages. Currently 80% of the world's population uses plant derived principles either directly or indirectly [1]. Certain examples of plant derived products employed as therapeutic agents are tannins, alkaloids, polyphenols, polysaccharides, essential oils, various extracts, and exudates. Lately, much research has been envisaged on polyphenols due to two main reasons. Firstly, these possess high spectrum of biological activities including antioxidant, anti-inflammatory, antibacterial, and antiviral and secondly, they are present in abundance in diet [2]. Polyphenols are found in many components of the human food including peanuts, dark chocolate, green and black tea, and turmeric. Extensive research in the past years and collected data shed light on certain physiological properties of plant polyphenols. These can slow the progression of certain cancers, neurodegenerative diseases, and diabetes and can reduce the risks of cardiovascular disease, thus highlighting the importance of the use of plant polyphenols as potential chemopreventive and anticancer agents in humans [3]. Many medicinal plants constitute polyphenols as active substances that modulate the activity of a wide range of enzymes and cell receptors [4]. However, the concentrations of polyphenols which appear effective *in vitro* are often of an order of magnitude lower than that required to elicit response *in vivo*, thus indicating their low bioavailability [5]. The bioavailability of polyphenols following oral administration is governed by many factors such as gastric residence time, permeability, and/or solubility within the gut. Further, the conditions encountered in food processing and storage (temperature, oxygen, and light) or in the gastrointestinal tract (pH, enzymes, and presence of other nutrients) may also influence the stability of polyphenols. Poor aqueous solubility and low dissolution rates of polyphenols contribute to their insufficient bioavailability [6]. There are two parameters that are useful for identifying a poorly soluble drug, the aqueous solubility and the dose: solubility ratio. A drug is classified as poorly soluble if it has less than 100 *μ*g/mL solubility. Dose: solubility ratio is defined as volume of gastrointestinal fluids necessary to dissolve the administered dose [7]. The majority of polyphenols belong to class II (low solubility and high permeability) and class IV (low solubility and low permeability) BCS classes thus limiting activity and potential health benefits of polyphenols. The bioavailability of class II and class IV substances may be enhanced by increasing the solubility and dissolution rate of the drug in the gastrointestinal fluid.  Table 1 depicts solubility and pharmacokinetic properties of some commonly used polyphenols [8–15]. The solubility of polyphenols can be enhanced by various techniques. Techniques used for improving solubility include inclusion complexes, micronization, solid dispersion, nanosuspension, solid lipid nanoparticles, nanostructured lipid carrier, liposomes, self- emulsifying drug delivery systems (SEDDS), and gel based systems.  Table 2 depicts some of commonly employed methods for increasing the solubility [16–23]. The present review discusses the various methods used till date to improve the bioavailability of polyphenols by enhancing their solubility.

## 2. Polyphenols: Types and Method for Solubility Enhancement

**Figure 1:**
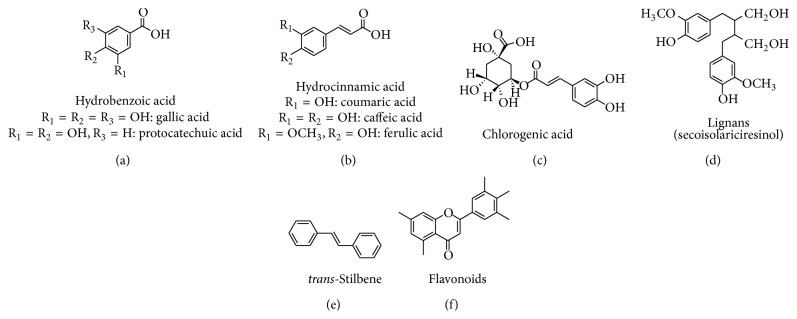
Chemical structure of polyphenols.

Several higher plants and some edible plants comprehend thousand molecules having a polyphenol structure (i.e., several hydroxyl groups on aromatic rings). These molecules are released as defense against ultraviolet radiation or aggression by pathogens and are a kind of secondary metabolites. The polyphenols are classified on the basis of the number of phenol rings that they contain and of the structural elements that bind these rings to one another. These are hence categorized into phenolic acids, flavonoids, stilbenes, and lignans.  Figure 1 depicts the chemical structure of polyphenols.

### 2.1. Phenolic Acids

They are plant derived phenolic compounds which are produced via shikimic acid through phenylpropanoid pathway and have a unique chemical structure of C_6_–C_3_. Some phenolic acids are also of microbial origin containing C_6_–C_1_ linkage. These are further classified into two categories: derivatives of cinnamic acid (hydroxycinnamic acids) and derivatives of benzoic acid (hydroxybenzoic acids).

#### 2.1.1. The Hydroxycinnamic Acids (Figure 1(b))

They are more common than the hydroxybenzoic acids and consist mainly of *p*-coumaric acid, caffeic acid, ferulic acid, and sinapic acid. These acids are found in glycosylated forms as derivatives of shikimic acid, quinic acid, and tartaric acid. Caffeic acid combines with quinic acid to form chlorogenic acid (Figure 1(c)). It is found in high concentrations in coffee: a single cup may contain 70–350 mg chlorogenic acid [24]. Caffeic acid is the most abundant phenolic acid and represents between 75 and 100% of the total hydroxycinnamic acid content of most of the fruit. All parts of the fruit contain hydrocinnamic acid but the highest concentrations are seen in the outer parts of ripe fruit. Cereal grains are dietary source of ferulic acid. Wheat grains may contain 0.8–2 g/kg dry weight of ferulic acid, which represents up to 90% of total polyphenols [25, 26]. Since, hydroxybenzoic acids possess sufficient aqueous solubility their absorption is not dissolution limited.

#### 2.1.2. Hydroxybenzoic Acids (Figure 1(a))

Salient examples of hydroxybenzoic acids are gallic acid, protocatechuic acid, ellagic acid (EA), and vanillic acid. Edible plants for example, red fruits, black radish, onions, and green tea are rich in hydroxybenzoic acid content [27]. Tea is an important source of gallic acid and tea leaves may contain up to 4.5 g/kg fresh wt of leaves [24, 28]. Dietary sources of EA include walnuts, pomegranates, and berries [29]. EA possesses several health benefits against many diseases such as breast cancer [30], prostate cancer [31], lung cancer [32], colon cancer [33], cardiovascular disease [34], and neurodegenerative diseases [35]. EA was found to possess maximum solubility of 9.3 *μ*g/mL [36]. This low solubility was attributed to high crystallinity of EA due to its planar and symmetrical structure and extensive hydrogen-bonding resulting in low bioavailability of EA. Solid dispersions of EA have been employed to enhance the solubility of EA. Li et al. [37] formulated solid dispersions of EA by three different methods.  Table 3 depicted the methods and compositions of these investigational formulations. Fourier transform infrared spectroscopy (FTIR) studies confirmed the presence of H-bonding between EA and polymers. Scanning electron microscope (SEM) studies indicated that EA was present in amorphous form in the solid dispersions. The *in vitro* dissolution studies revealed that the nature of polymer directly influences the solubility of EA. The polymer with more hydrophilic character resulted in higher swelling and faster release of EA. Thus, the release profile of EA from EA/PVP matrix was 92% (1 h) followed by EA/HPMCAS (35%, 0.5 h), EA/CMCAB (18%, 1 h), and EA/CAAdP (15–17%, 1 h). Incorporation of CAAdP in EA/PVP solid dispersion led to a decrease in release of EA (62%, 0.5 h). EA has been reported to deteriorate in the solution form due to crystallization and chemical degradation. The amount of EA remaining after 24 h in solution is only 18% and 80% due to crystallization and chemical degradation, respectively. However, the solid dispersions were found to significantly enhance the stability of EA against crystallization and chemical degradation. Further, it was found that HPMCAS amorphous solid dispersion provided maximum stability to EA [37].

### 2.2. Flavonoids

**Figure 2:**
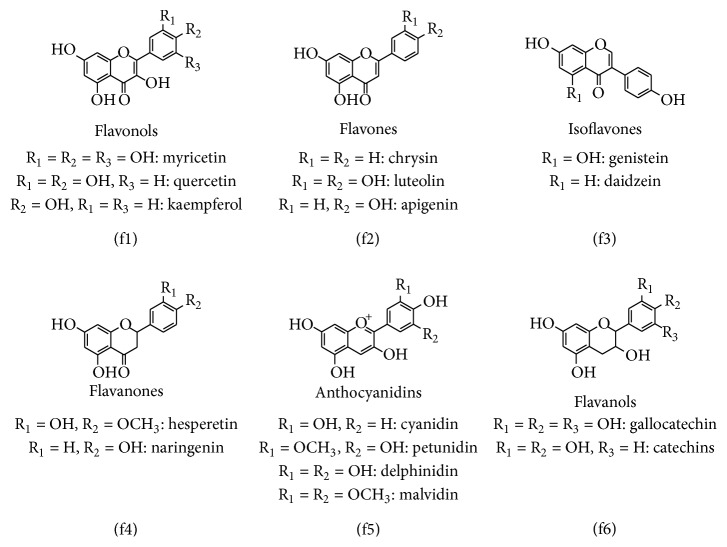
Classification and chemical structure of flavonoids.

These are benzo-*γ*-pyrone derivatives of phenolic and pyran rings [38]. On the basis of substitutions on three rings, flavonoids are classified as flavonols, flavones, isoflavones, flavanones, flavanols, and anthocyanidins which are biotransformed in body by methylation, sulfation, and glucuronidation of hydroxyl groups. Flavonoids predominantly exist as 3-O-glycosides and polymers [39]. Chemical structure of flavonoids is illustrated in Figure 2.

#### 2.2.1. Flavonols

Among flavonoids, *flavonols* are the most ubiquitous in foods. The main representatives of flavonols are quercetin and kaempferol (Figure 2(f1)). The flavonols are primarily found in onions (up to 1.2 g/kg fresh wt), curly kale, leeks, broccoli, and blueberries. Red wine and tea also contain up to 45 mg/L flavonols. In nature, flavonols are present in glycosylated forms in plants. The sugar moiety associated with flavonols is mainly glucose or rhamnose, but other sugars like galactose, arabinose, and xylose may also be involved. Each fruit contains around 5–10 different flavonol glycosides [40]. The biosynthesis of flavonols is stimulated by light, so these tend to accumulate in the outer and aerial tissues. Depending on exposure to sunlight, differences in concentration exist between fruits on the same tree and even between different sides of a single fruit [41].

*Quercetin.* It is a naturally occurring polyphenol which belongs to a group of plant pigments known as flavonoids, responsible for the colour of vegetables, fruits, and flowers [42]. Quercetin is a flavonol whose chemical structure is derived from flavone. Chemically quercetin is known as 3,3,4′,5,7-pentahydroxyflavone. Quercetin exhibits various properties such as anti-inflammatory, antioxidant, antihistamine, and antiarthritis [42]. The primary dietary sources of quercetin are citrus fruits, apple, onions, parsley, sage, tea, and red wine [43]. However, despite having all these beneficial activities, poor water solubility (0.3 *μ*g/mL) restricts its use thus highlighting the importance of increasing the solubility of quercetin [44]. Gao et al. [45] reported the formation of nanosuspension of quercetin by two techniques. The first technique is comprised of evaporative precipitation of quercetin into aqueous solution (EPAS). The organic solution of quercetin in ethanol was poured slowly into an aqueous solution containing Pluronic F68 (0.75% w/v) and lecithin (0.25% w/v) stabilizers. The solution was continuously stirred under vacuum. Finally, ethanol was evaporated and EPAS nanosuspension was collected. The second technique involved high pressure homogenization (HPH) of quercetin dispersion in Pluronic F68 (0.75% w/v) and lecithin (0.25% w/v). A piston gap high pressure homogenizer was used to circulate the suspension for two cycles at the pressure of 200 bar and five cycles at 500 bar followed by 20 more cycles at 1500 bar resulting in HPH suspension. The mean particle size, polydispersity index (PI), and solubility profile of quercetin nanosuspension produced by EPAS method and HPH method were found to be 282.6 ± 50.3 nm, 0.23 ± 0.08, 422.4 *μ*g/mL and 213.6 ± 29.3 nm, 0.21 ± 0.10, 278.6 *μ*g/mL, respectively. X-ray powder diffraction (XRPD) measurements revealed a crystalline to amorphous phase transition in EPAS process, which was not observed in HPH. This formed the basis for higher increase in solubility of quercetin in case of EPAS [46].

A solid dispersion of quercetin employing CMCAB, HPMCAS, and CAAdP as polymers has been reported by Li et al. [47]. Quercetin and polymer mixtures were prepared in different ratios of 1 : 9, 1 : 3, 1 : 1, 3 : 1, and 9 : 1. Acetone : ethanol (1 : 4) solution was used to dissolve the above mixtures to form 2% w/v solution. The solutions were spray-dried using inlet temperature 90°C, outlet temperature 57–60°C, feed rate 9 mL/min, and nitrogen flow rate 350 L/h. XRPD studies of the formulations revealed that while quercetin/CMCAB had identical crystallinity, quercetin/CAAdp showed amorphous character and quercetin/HPMCAS displayed partial crystalline character with respect to crude quercetin. FTIR spectra of the formulations showed broadening of peak at 3300–3500 cm^−1^ which was attributed to the presence of intermolecular H-bonding between quercetin and matrix polymer, further decreasing crystalline structure of quercetin. A comparison of release profiles of quercetin solid dispersion with quercetin powder indicated that the solid dispersions quercetin/HPMCA, quercetin/CMCAB, and quercetin/CAAdp showed 14% release after 0.5 h whereas the dissolution of quercetin powder was found to be 0.7% even after 1 h.

Inclusion complexes of quercetin have also been reported for increasing solubility of quercetin [48]. An inclusion complex of quercetin was made with *β*-cyclodextrin (*β*CD), hydroxypropyl-*β*-cyclodextrin (HP-*β*CD), and sulfobutyl ether-*β*-cyclodextrin (SBE-*β*CD) (ranging from 0 to 0.01 M). The 1 : 1 complex between quercetin and cyclodextrins led to increased solubility of quercetin in the order of *β*CD < HP-*β*CD < SBE-*β*CD [49].

#### 2.2.2. Flavones

They are much less prevalent than flavonols in fruit and vegetables. Flavones primarily contain glycosides of luteolin and apigenin (Figure 2(f2)). The important savory sources of flavones are parsley and celery. *C*-glycosides of flavones are encompassed in cereals such as millet and wheat [50, 51].

*Apigenin (AP).* It is a naturally occurring flavone chemically known as 4′,5,7-trihydroxyflavone. The most prevalent natural sources of AP are parsley, celery, and chamomile tea [52]. AP belongs to BCS class IIwith poor aqueous solubility and high permeability in intestine. AP was found to possess maximum solubility 2.16 *μ*g/mL at pH 7.5 resulting in low dissolution and poor bioavailability [53]. Various formulation strategies have been devised to overcome this problem. High shear mixing for preparation of AP smart crystals has been reported by Al Shaal et al. [54] for solubility enhancement of AP.

Smart crystal technology comprehends combination of different processes, pretreatment of poorly soluble drug followed by high pressure homogenization. A macrosuspension of AP powder and surfactant solution (Plantacare 2000 UP, 1% w/w) was formed by high shear mixing (Ultra-Turrax T25, 10,000 rpm). This was followed by seven passages through bead milling (Buhler PML-2). The formed nanosuspension was then subjected to high pressure homogenization (Avestin C50, 300 bar/cycle). The pretreatment step was included to accelerate nanocrystals production by reducing homogenization cycles and to decrease particle size. Milling medium used was zirkonia and yttria was employed as a stabilizer. The mean particle size of AP was found to be 439 ± 20 nm with a low PI of 0.283 ± 0.040. Light microscopy studies also presented evidence supporting the use of surfactant by showing an image with uniform crystal distribution with no signs of large crystals and aggregates in the presence of surfactant. A zeta potential of −38 mV was reported which indicated a well charged surface and related stability. AP coarse powder and nanoparticles showed identical X-ray diffraction (XRD) pattern indicating no decrease in crystallinity. DPPH (2,2-diphenyl-1-picrylhydrazyl) radical scavenging test showed a 2-fold increase in antioxidant activity of AP nanoparticles as compared to AP macrosuspension.

**Figure 3:**
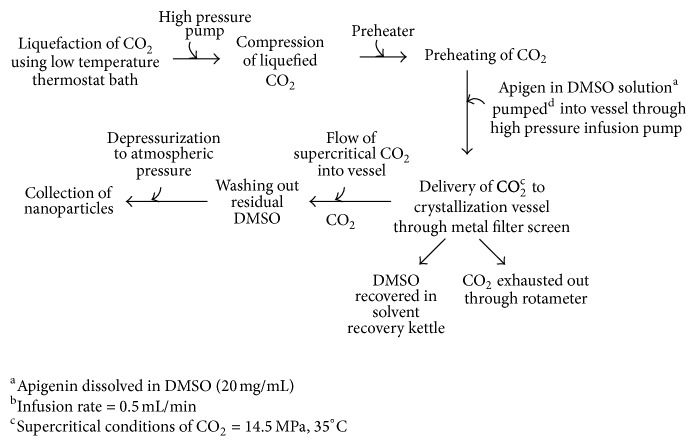
Steps involved in SAS method for preparation of nanoparticles [55].

Another method for improvement in solubility of AP has been reported by Zhang et al. [55]. The study incorporated preparation of AP nanocrystals via supercritical antisolvent method (SAS).  Figure 3 depicts a schematic representation of preparation of nanoparticles. Photon correlation spectroscopy (PCS) studies revealed the particle size to be 562.5 ± 56 nm with a PI value of 0.92 ± 0.21. Reduced degree of crystallinity was represented in XRPD diagram. Differential scanning calorimetry (DSC) curves of AP coarse powder and AP nanocrystals were studied and compared. A decrease in melting point of AP was observed with nanoparticles which could be attributed to particle size reduction to nanometer range. FTIR patterns were identical for both coarse powder and nanoparticles thus indicating the chemical stability of AP during SAS process. AP nanocrystals exhibited more rapid dissolution rate with much higher cumulative amount of dissolved AP than AP coarse powder. The higher dissolution of AP nanocrystals could be due to the enhanced saturated solubility resulting from a significant decrease of particle size [56]. *In vivo* studies showed 3.6 and 3.4 fold enhancement in *C*_max⁡_ and AUC of AP, respectively, after oral administration of AP nanocrystals. The absolute bioavailability of AP coarse powder was found to be 2.0% whereas nanoparticles exhibited 6.9% absolute bioavailability. Thus improved solubility, dissolution rate, and bioavailability depict the usefulness of these methods for delivery of such BCS class second compounds.

#### 2.2.3. Flavanones

They are natural compounds of restricted occurrence and are sometimes termed as minor flavonoids. The cardinal aglycones are naringenin, hesperetin, and eriodictyol (Figure 2(f4)). Glycosylation of flavanones is generally attained by a disaccharide at position 7, which is either a neohesperidose, that imparts a bitter taste (such as to naringin in grapefruit), or a rutinose, which is flavorless. Citrus fruits contain considerable amount of flavanones. Tomatoes and certain aromatic plants such as mint also constitute flavanones. Hesperidin and narirutin are present in orange juice at a concentration of 200–600 mg/L and 15–85 mg/L, respectively. A single glass of orange juice may contain between 40 and 140 mg of flavanone glycosides [57]. However, very high flavanone content is found to be present in the solid parts of orange fruit, particularly the albedo (the white spongy portion), and the membranes separating the segments. Thus an orange fruit may comprise up to 5 times as much as a glass of orange juice.

*(1) Hesperetin.* It is a naturally occurring flavonoid chemically known as 3′,5,7-trihydroxy-4-methoxyflavanone. Hesperetin is found almost exclusively in citrus fruits [58]. Studies revealed that hesperetin can avert colon [59], urinary bladder [60], and chemically induced mammary carcinogenesis [61]. Other biological activities of hesperetin include antioxidant [62] and anti-inflammatory [63]. Aqueous solubility of hesperetin was found to be 1.4 *μ*g/mL [64]. Nanoparticles of hesperetin by two different methods namely APSP and EPN have been have been reported to enhance the solubility and dissolution rate [65].

*APSP.* The solvent and antisolvent used in this method were ethanol and deionized water, respectively. The method comprised of dissolution of hesperetin in solvent followed by injection of drug solution into an antisolvent with the help of syringe. The solution was constantly stirred using magnetic stirrer (200–1000 rpm) and the flow rate was varied from 2 to 10 mL/min.

*EPN.* Nanoparticles in this method were formed by quick addition of drug solution containing hesperetin and ethanol into antisolvent. Hexane was used as antisolvent. Vacuum drying was carried out for quick evaporation of solvent leading to formation of nanosuspension.

**Figure 4:**
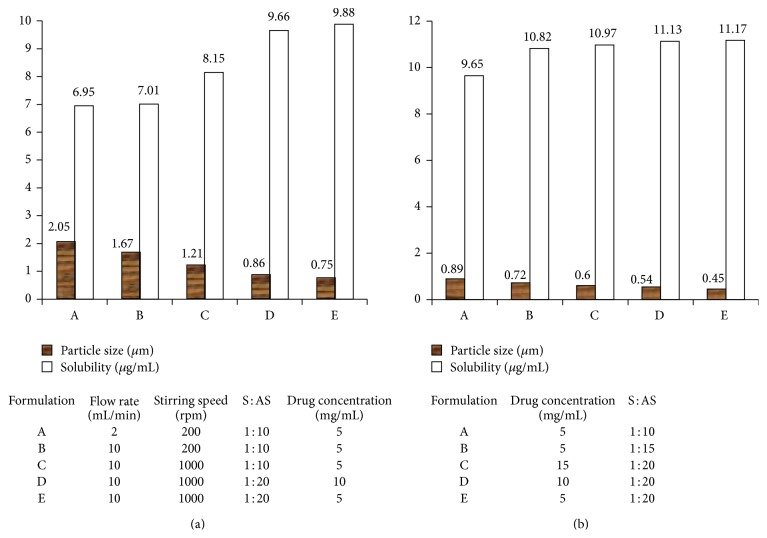
(a) Depicting the effect of various parameters on particle size and solubility of hesperetin by APSP method [65]. (b) Depicting the effect of various parameters on particle size and solubility of hesperetin by EPN method [65].

Figures 4(a) and 4(b) depict the effect of various parameters on particle size and solubility of hesperetin by APSP and EPN methods. The formulation containing 5 mg/mL drug concentration, 10 mL/min flow rate, stirring speed of 1000 rpm, and solvent : antisolvent ratio of 1 : 20 depicted highest solubility (9.88 *μ*g/mL). This was attributed to a decrease in particle size from 34 *μ*m to 0.75 *μ*m (revealed by SEM). In case of EPN highest solubility (11.17 *μ*g/mL) was seen in formulation containing 5 *μ*g/mL drug concentration and 1 : 20 solvent : antisolvent ratio. The DSC studies revealed that the melting point of nanoparticles prepared by both methods was identical to crude hesperetin but enthalpy of fusion was reduced due to reduction in crystallinity of nanoparticles of hesperetin.

*(2) Naringenin (NRG).* It is a kind of flavanone (4′,5,7-trihydroxyflvanone), found extremely in tomatoes [66], cherries [67], grape fruit, and citrus fruits [58]. In addition to antioxidant property [68], NRG also possess anti-inflammatory [69], antitumour [70], and hepatoprotective effects [71]. However, clinical applicability of NRG is limited by its low solubility and bioavailability. NRG possesses low aqueous solubility (45 *μ*g/mL) [72], therefore measures were taken to investigate methods for enhancing solubility of NRG. Transglycosylation of hesperetin leads to an increase in solubility of hesperetin [73]. This formed the basis for preparing spray-dried particles of NRG with *α*-Glucosyl hesperidin (Hsp-G) in order to enhance its solubility [72]. Different loading ratios of NRG/Hsp-G (1 : 1 to 1 : 20 w/w) were dissolved in ethanol : water (8 : 2 v/v) solution. The resultant suspension was then subjected to spray drying at the rate of 10 mL/min employing a spray nozzle of diameter 406 *μ*m and pressure of 0.13 MPa. The inlet and outlet temperatures of drying chamber were 120°C and 70°C, respectively. SEM images of NRG/Hsp-G samples showed spherical shaped aggregates with average particle size of 3-4 *μ*m. The resultant spray-dried particles of NRG showed 60-fold improvement in solubility when loading ratio of NRG/Hsp-G was 1 : 20.

#### 2.2.4. Isoflavones

They have structural similarities to estrogens as they have hydroxyl groups in positions 7 and 4′ in a configuration analogous to that of the hydroxyls in the estradiol molecule (Figure 2(f3)). Although isoflavones are not steroids, they have potential estrogenic activity. This illustrates their ability to bind to estrogen receptors. They possess pseudohormonal properties and are consequently classified as phytoestrogens [2]. Leguminous plants are the exclusive source of isoflavones. The main source of isoflavones in the human diet is soya and its processed products. Isoflavones principally contain 3 compounds: genistein, daidzein, and glycitein (concentration ratio of 1 : 1 : 0.2). Factors such as geographic zone, growing conditions, and processing of soya and its manufactured products greatly affect their isoflavone content. Isoflavone content of soybeans is 580–3800 mg/kg and of soymilk is 30–175 mg/L [74, 75].

*Genistein.* It is a naturally occurring plant flavonoid. Soy products are the richest sources of genistein [76]. Chemical structure of genistein (4′,5,7-trihydroxyisoflavone) contains an isoflavone backbone. Genistein has beneficial effects in areas of cancer [77], cardiovascular diseases [78], and postmenopausal symptoms [79]. Aqueous solubility of genistein is very poor, approximately 0.81 *μ*g/mL [80], which leads to low bioavailability of the drug. A solid dispersion of genistein in Pluronic F127 polymeric micelles has been reported for solubility enhancement by Kwon et al. [80]. An ethanolic solution of Pluronic F127 was used to dissolve genistein by constant stirring at 37°C for 30 min. The solution when evaporated led to formation of clear gel-like matrix. Addition of water and constant stirring resulted in formation of polymeric micelles containing genistein. The resulting solution was filtered employing 0.45 *μ*m pore size membrane filter to remove any undissolved genistein followed by lyophilization at −80°C. Average particle size of genistein loaded polymeric micelles was found to be 27.76 ± 0.46 nm with PI of 0.26. *In vitro* drug release showed genistein release 48–58% in pH 1.2 medium and 44–82% in pH 6.8 medium which was attributed to higher solubilizing ability of polymeric micelles. The *in vivo* pharmacokinetic characterization showed an increase in *C*_max⁡_ from 1.22 to 5.68 *μ*g/mL and decrease in *t*_max⁡_ from 0.55 to 0.20 h. The polymeric micelles also demonstrated enhanced bioavailability thus confirming enhanced genistein solubility and release in gastrointestinal tract.

### 2.3. Stilbenes

Stilbenes encompass a group of biologically active compounds; however, human diet comprises only few of these (Figure 1(e)). Examples may include trans-resveratrol and trans-piceid (its natural glycoside).

#### 2.3.1. Resveratrol

It belongs to a class of naturally occurring polyphenols known as stilbenes. It is mainly present in the form of *trans*-resveratrol (3,5,4′-trihydroxystilbene) in human diet. The dietary sources of resveratrol include peanut butter, dark chocolate, blueberries, and red wine. About 2.3 mg/L of trans-resveratrol is present in red wine [81]. Resveratrol exhibits antiangiogenesis [82], cardioprotective [83], anticarcinogenic, and anti-inflammatory activities [84]. Aqueous solubility of resveratrol was found to be 30 *μ*g/mL, thereby, limiting pharmaceutical potential of resveratrol [85]. Zhang et al. [86] reported a method for enhancing solubility of resveratrol by formulating nanoparticles of resveratrol using antisolvent precipitation method. The ethanolic solution of resveratrol (solvent) was poured with vigorous stirring (9000 rpm) into aqueous solution of polymer (antisolvent) resulting into precipitation of resveratrol after 30 s. Four different polymers that is, HPMC, PVP, PEG 400, and P188 were employed. The solvent : antisolvent ratio was kept constant at 1 : 20. Process parameters employed for spray drying were 105°C inlet temperature, 50–60°C outlet temperature, 1 mL/min spray flow rate, and 0.65 MPa atomization air pressure. The particle size obtained with HPMC, PVP, PEG 400, and P188 was found to be 161 ± 3, 1156 ± 78, 2168 ± 26, and 1644 ± 47 nm, respectively. Dissolution studies represented complete dissolution of resveratrol nanodispersion in less than 45 min, whereas raw resveratrol did not dissolve completely even after 120 min indicating increased water solubility of resveratrol by using polymers.

### 2.4. Miscellaneous

#### 2.4.1. Curcumin

**Figure 5:**
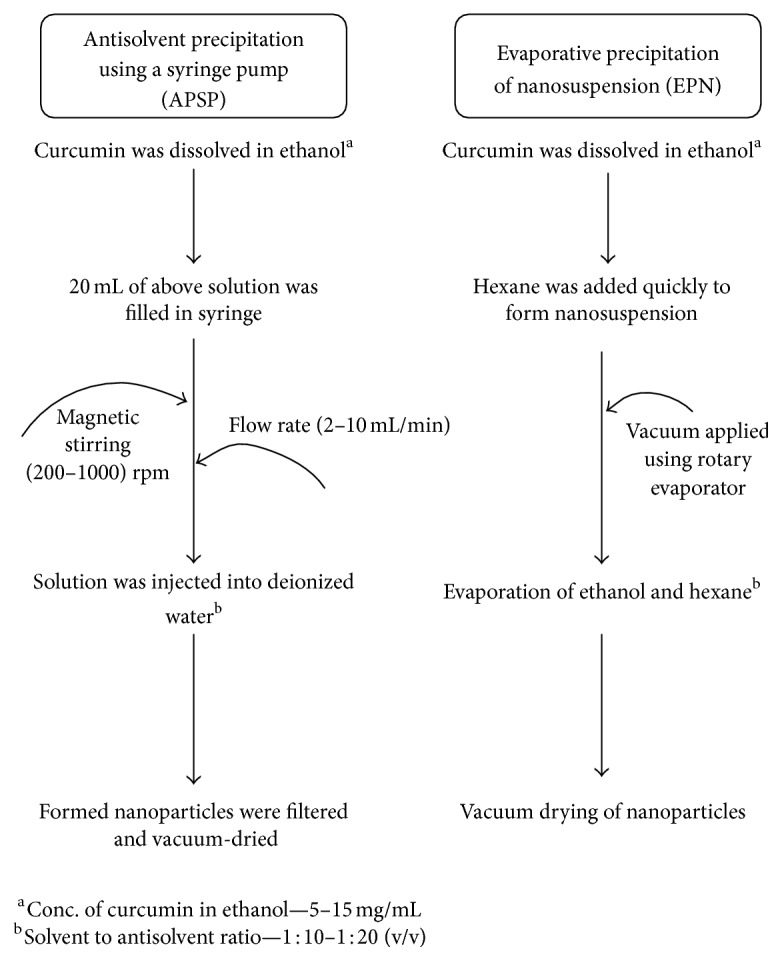
APSP and EPN techniques for nanoparticle formulation of curcumin [92].

It is a naturally occurring polyphenol which is extracted from the plants of *Curcuma longa. Curcuma longa* (turmeric) has been used to treat ailments since a long time ago. It is also employed as a spice in Indian cuisine. Curcumin exhibits a variety of pharmacological actions such as antitumor [87], anti-HIV [88], antioxidant, and anti-inflammatory [89]. However, the goodness of curcumin has not been able to reach up to its potential yet. The maximum solubility of curcumin in plain aqueous buffer pH 5.0 has been reported to be 11 ng/mL [90] and the oral dose of curcumin for treating advanced colorectal cancer was found to be 3.6 g/day [91]. Therefore, there is need to devise strategies to increase solubility of curcumin. Nanoparticles of curcumin employing antisolvent precipitation method have been reported by Kakran et al. [92]. The antisolvent precipitation involved two methods, namely, antisolvent precipitation using a syringe pump (APSP) and evaporative precipitation of nanosuspension (EPN). In first method ethanol was used as solvent and deionized water as antisolvent. In EPN method solvent was same but antisolvent employed was hexane.  Figure 5 depicts a schematic representation of techniques employed for formulation of nanoparticles. The effect of process variables such as stirring speed, flow rate, solvent : antisolvent (S : AS) ratio, and drug concentration was studied on particle size and solubility.

An increase in the stirring speed from 200 to 1000 rpm in APSP leads to a decrease in particle size from 550 to 500 nm. An increase in stirring speed led to intensification of micromixing between multiphases resulting in decrease in particle size. Similar results were observed with a variation in flow rate of curcumin solution. An increase in the flow rate from 2 to 10 mL/min led to decrease in length of curcumin particles from 2560 to 1860 nm since an increase in flow rate resulted in rapid mixing. Further, an inverse relationship was reported between amount of antisolvent in SAS ratios and particle size. With an increase in S : AS ratio 1 : 20 from 1 : 10, a decrease in length and diameter of curcumin particles from 1860 and 490 nm to 930 and 340 nm, respectively, was reported. The drug concentration exhibited a direct relationship with particle size as greater supersaturation followed by faster nucleation rate and smaller particles was observed with an increase in drug concentration. The particle length increased from 930 to 965 nm with a change in drug concentration from 5 to 15 mg/mL. Further, DSC studies revealed a decrease in curcumin crystallinity owing to decrease in melting enthalpy of nanoparticles although melting point was identical to original curcumin. The solubility studies indicated that solubility of curcumin (0.58 ± 0.03 *μ*g/mL) was increased by APSP and EPN methods to 7.48 ± 0.11 *μ*g/mL and 8.23 ± 0.07* μ*g/mL, respectively, which was ascribed to a reduced particle size [93] and decreased crystallinity [46].

**Figure 6:**
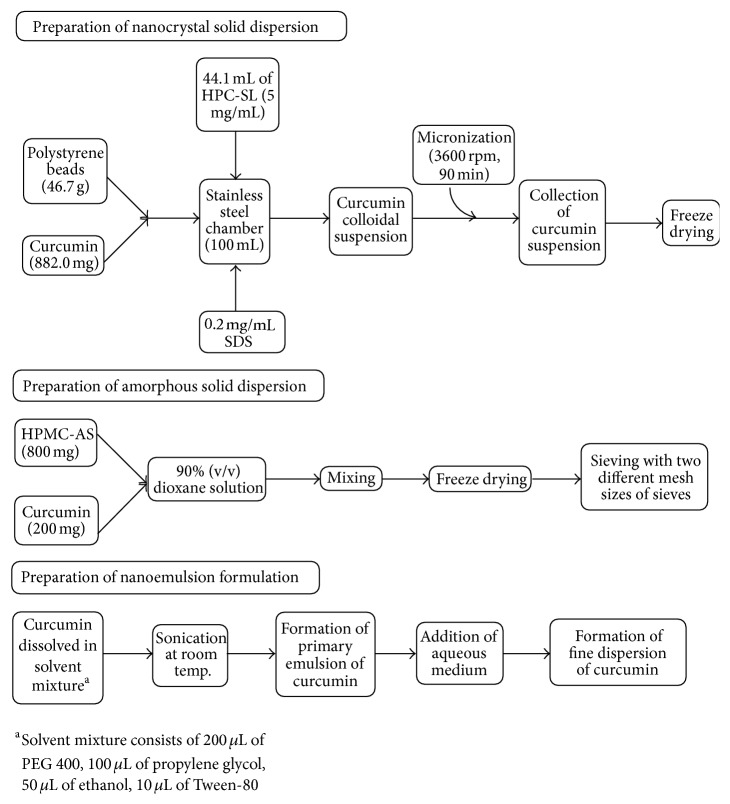
Formulation of curcumin solid dispersions [94].

*Nanocrystal Solid Dispersions of Curcumin.* Solid dispersions have also been demonstrated to increase the bioavailability of poorly water soluble drugs. Onoue et al. [94] have reported the formulation of solid dispersions of curcumin to enhance its solubility. Three types of curcumin dispersions were formulated, namely, nanocrystal solid dispersion (CSD), amorphous solid dispersion (ASD), and nanoemulsion (NE).  Figure 6 shows the schematic representation of preparation of three types of solid dispersions. Diffraction pattern of CSD-curcumin was identical with crystalline curcumin indicating high crystallinity of curcumin whereas ASD-curcumin was found to be amorphous. Release rates of amorphous solid dispersion, nanocrystal solid dispersion, and nanoemulsion formulation were found to be 95% (180 min), 80% (180 min), and 93% (60 min), respectively, thus, indicating enhanced solubility of curcumin with solid dispersions.

*Self-Microemulsifying Drug Delivery Systems of Curcumin (SMEDDS).* Use of SMEDDS as one of the approaches to enhance solubility, dissolution, and oral absorption of poorly water soluble drugs has gained interest recently [95]. Cui et al. [96] employed SMEDDS for enhancing solubility of curcumin. Curcumin loaded SMEDDS were formulated employing oil (ethyl oleate), surfactant (the mixtures of emulsifier OP : cremorphor EL-40 1 : 1 w/w), and cosurfactant (PEG 400). Different concentrations of the three components were used and evaluated for particle size and solubility. The optimal concentration of oil, surfactant, and cosurfactant was found to be 12.5%, 57.5%, and 30%, respectively. According to transmission electron microscopy (TEM) images, the mean particle size of formulation after dilution with water was found to be 21 nm and the solubility of curcumin was enhanced to 21 mg/g. A rapid dissolution (85% in 10 min) was observed with SMEDDS whereas crude curcumin showed negligible release even after 60 min in both pH 1.2 and 6.8 buffer solutions. *In vivo* oral absorption of curcumin loaded SMEDDS depicted 3.8-time increase in absorption percentage of SMEDDS.

## 3. Conclusion

Increase in solubility of a therapeutic agent can enhance the bioavailability of that compound. Polyphenols are naturally occurring active principles with wide variety of physiological and biological activities. However, their therapeutic potential has not been exposed widely because of their low solubilities. This review discusses the various techniques employed so far for solubility enhancement of polyphenols. The different strategies for example, antisolvent precipitation, evaporative precipitation, high pressure homogenization, or SMEDDS resulted in approximately 15–20-fold enhancement in solubility and 3–5-fold enhancement in bioavailability for some polyphenols, thus suggesting that application potential of polyphenols can be enhanced by increasing their solubility.

## Figures and Tables

**Table 1:** Pharmacokinetic properties of polyphenols.

Polyphenol	Solubility (*µ*g/mL)	Dose (*µ*M)	*C*_max⁡_ (*µ*M)	*T*_max⁡_ (h)	References
Phenolic acid					
Ellagic acid	9.3	44.67	0.036	1.98	[8, 36]
Stilbenes					
Resveratrol	30	109.5	0.031	0.5	[9, 85]
Flavonols					
Quercetin	0.3	255	0.74	0.7	[10, 44]
Flavones					
Apigenin	2.16	65.8	0.12	7.2	[11, 53]
Flavanones					
Hesperetin	1.4	727	1.3	5.8	[12, 64]
Naringenin	45	166	0.2	5.0	[12, 72]
Anthocyanins					
Cyanidin-3-rutinoside	—	137	0.05	1.5	[13]
Delphinidin-3-rutinoside	—	182	0.07	1.8	[13]
Isoflavones					
Genistein	0.81	70	0.75	6.5	[14, 80]
Daidzein	8.215	98	0.79	6.5	[14, 15]

**Table 2:** Strategies to improve solubility of polyphenols.

Method	Procedure	Advantages	Disadvantages	Example	Reference
Nanoparticles					[16–18]

Evaporative precipitation into aqueous solution	Spraying of drug solution through an atomizer into an aqueous solution containing stabilizer at high temperature.	High dissolution rate, high surface area, enhanced wettability.	Require stabilizers, lack of controlled release, not suitable for thermolabile drugs	Nanosuspension of quercetin	[45]

High pressure homogenization	Precipitation of drug by addition of antisolvent in the drug solution leading to formation of unstable form of drug which is stabilized by means of single/repeated application of high energy followed by thermal relaxation (annealing).	Reduced particle size, enhanced dissolution, no crystal growth	Long processing time, introduction of impurities, high energy requirements, chemical degradation	Nanosuspension of quercetin	[45]

Antisolvent method					
Antisolvent precipitation using a syringe pump	Addition of antisolvent to a solution of drug and solvent at a particular flow rate under constant stirring leading to precipitation of drug which is then filtered to collect nanoparticles.	Reduced particle size, high dissolution rate, high surface area, reduced crystallinity, faster onset of action	Contamination due to filtration	Nanoparticles of hesperetin	[65]
Evaporative precipitation of nanosuspension	Mixing of a water miscible solvent containing drug with an antisolvent followed by evaporation of solvents.	Decreased particle size, enhanced surface area, improved dissolution	Particle growth due to remaining organic solvent in suspension	Curcumin nanoparticles	[92]
Supercritical antisolvent method	Precipitation of drug from drug solution by mixing it with a compressed fluid at its supercritical conditions. Diffusion of solvent into antisolvent phase leads to drug precipitation due to low solubility of drug in antisolvent.	High product purity, controlled crystal polymorphism, possible processing of thermolabile molecules, single step process	Toxicity and flammability of solvents, poor control of particle morphology, incomplete removal of residual solvent	Apigenin nanocrystals	[55]

Solid dispersion	Formation of eutectic mixtures of drugs with hydrophilic carriers by melting their physical mixtures	Particle size reduction, improved wettability, enhanced dissolution, higher porosity	Decrease in dissolution on aging, crystal growth upon moisture absorption, demixing, phase separation	Solid dispersion of ellagic acid	[19–21, 37]

Self-microemulsifying drug delivery systems	Gentle mixing of drug, oil, surfactant, and cosurfactant in aqueous media leading to formation of o/w microemulsion of drug droplets with mean droplet size <100 nm.	Higher bioavailability, improved absorption, oral administration using gelatin capsules	Surfactant toxicity, tedious manufacturing method, interaction with capsule shell	Curcumin	[22, 23, 96]

**Table 3:** Different methods for solid dispersions of ellagic acid [37].

Method	Composition	Procedure
Spray-dried solid dispersion	Acetone : ethanol (1 : 4 v/v) solution, ellagic acid, polyvinylpyrrolidone (PVP), carboxymethyl cellulose acetate butyrate (CMCAB), hydroxypropyl methyl cellulose acetate succinate (HPMCAS)	Acetone : ethanol solution was used to dissolve mixtures of EA/polymer followed by spray drying of the resultant dispersion under operating conditions of 90°C inlet temperature, 57–60°C outlet temperature, 9 mL/min feed rate, and 350 L/h nitrogen flow.

Coprecipitated solid dispersion	Ellagic acid, tetrahydrofuran (THF), cellulose acetate adipate propionate (CAAdP)	A mixture of EA/CAAdP was dissolved in THF followed by dropwise addition of the solution in deionized water with stirring.

Solid dispersion by rotary evaporation	Ellagic acid (20 mg), PVP (90 mg), CAAdP (90 mg), acetonitrile : ethanol (1 : 1 v/v) solution (40 mL)	EA, PVP, and CAAdP were dissolved in acetonitrile : ethanol solution followed by concentrating the solution with rotary evaporation

## Abbreviations

AP: Apigenin
APSP: Antisolvent precipitation using a syringe pump
ASD: Amorphous solid dispersion
*β*-CD: *β*-Cyclodextrin
BCS: Biopharmaceutics classification system
CAAdP: Cellulose acetate adipate propionate
CSD: Nanocrystal solid dispersion
CMCAB: Carboxymethyl cellulose acetate butyrate
DPPH: 2,2-Diphenyl-1-picrylhydrazyl
EA: Ellagic acid
EPAS: Evaporative precipitation of nanosuspension
EPN: Evaporative precipitation of nanosuspension
HP-*β*-CD: Hydroxypropyl-*β*-cyclodextrin
HPH: High pressure homogenization
HPMC: Hydroxypropyl methyl cellulose
HPMCAS: Hydroxypropyl methyl cellulose acetate succinate
NRG: Naringenin
NE: Nanoemulsion
PEG: Polyethylene glycol
PVP: Polyvinylpyrrolidone
SAS: Supercritical antisolvent method
SBE-*β*-CD: Sulfobutyl ether-*β*-cyclodextrin
THF: Tetrahydrofuran.
